# Inhibition of HIV-1 Protease by *Carpobrotus edulis* (L.)

**DOI:** 10.1155/2020/9648056

**Published:** 2020-06-07

**Authors:** Beauty E. Omoruyi, David I. Ighodaro, Anthony J. Afolayan, Graeme Bradley

**Affiliations:** ^1^Applied Microbial and Health Biotechnology Institute, Cape Peninsula University of Technology, P.O. Box 1906, Bellville 7535, Cape Town, South Africa; ^2^Department of Agricultural Extension, Cape Peninsula University of Technology, P.O. Box 1906, Bellville 7535, Cape Town, South Africa; ^3^Medicinal Plant and Economic Development Research Centre, Department of Botany, University of Fort Hare, Private Bag X1314, Alice 5700, South Africa; ^4^Department of Biochemistry and Microbiology, University of Fort Hare, Private Bag X1314, Alice 5700, South Africa

## Abstract

*Carpobrotus edulis* (L.) is a plant commonly found in the Eastern Cape Province of South Africa and is used for the general treatment of infections relating to the human immunodeficiency virus (HIV). HIV-1 protease plays an important role during HIV replication and maturation to its infectious form, and therefore inhibition of the enzyme is one of the main focus areas in drug development. The inhibitory effect of a water extract of *C. edulis* leaves against HIV-1 protease activity was determined using the SensoLyte® 520 HIV-1 protease assay fluorimetric kit and employing a HiLyte Fluor™488/QXL™520 fluorescence resonance energy transfer (FRET) peptide. Cytotoxicity of the extract towards HeLa Chang cell lines was determined using an *in vitro* MTT assay, and the phytochemical profile of the extract was determined with FT-IR and LC-MS. HIV-1 protease activity was inhibited 83.06% (IC_50_ 1.6 mg/ml) (*p* < 0.0001) by the pepstatin A inhibitor control. Treatment with all *C. edulis* extract concentrations (16, 1.6, 0.16, and 0.016 mg/ml) inhibited HIV-1 protease activity significantly (*p* < 0.0001) in a typical dose response manner. With regards to cytotoxicity, the negative controls containing untreated HeLa Chang cells exhibited high formazan formation rates in contrast with the positive controls, containing curcumin, which reduced formazan formation significantly (*p* < 0.001), exhibiting cytotoxicity towards the cells. There was no significant (*p* > 0.05) difference in the formazan formation rates between the negative controls and 1, 0.5, 0.125, 0.065, 0.031, and 0.015 mg/ml plant extract, confirming no toxicity of *C. edulis* extracts towards HeLa Chang cells. Major functional phytochemical compounds identified included alcohols, phenols, alkanes, amines, carboxylic acids, and esters. LC-ESI-TOF/MS analysis revealed the putative identities of main compounds present in the aqueous leaves extract, including some that contribute to its anti-HIV-1 protease action.

## 1. Introduction

The maturation of the human immunodeficiency virus (HIV) to its infectious form is dependent on the posttranslational cleavage of virus Gag (p55) and Gag-Pol (p160) structural proteins by HIV-1 protease [1]. HIV-1 protease is an aspartic protease with a molecular weight of 21.6 kDa. It is a dimeric enzyme composed of 99 amino acids residues of Asp25-Thr26-Gly27-Asp30 at the catalytic site [2]. The active site loops are held together by a network of hydrogen bonds between the active site amino acids and the surrounding residues [2]. Due to the strong hydrogen-bonding forces between the Thr26 residues, the conformational core domain of the HIV-1 protease becomes very stable [3]. Amino acid residues of Gly27 accommodate and bind a substrate in a corresponding position in which the catalytic Asp25 carboxylate groups can attack the amide moiety of a substrate [4]. Also, the active site is covered by two extended beta-hairpins known as flap residues (lle47-Gly48-Gly49-lle50), which bind firmly to other polyproteins of Phe-Pro, Pro-Tyr, and Leu-Phe, enhancing multiplicity of the virus [5]. Consequent to the manipulative cleavages of amino residues within the host cell protein, the virus thus complicates its initial recognition by CD4 receptors and T-cells [6].

Multimerization of polyproteins residues along with complex binding of the viral genomic RNA and other viral proteins in the host cell system gives rise to the formation of new virion particles [7]. The internalization of the newly formed virus is typically triggered by structural protein matrix A (MA), capsid (CA), and nucleocapsid (NC); this includes other spacer peptides p1, p2, and p6 at the virus catalytic site [8]. During the late phase of the virus fusion, the myristoylated N-terminal MA protein directs Gag-Pol towards the plasma membrane, where the NC proteins associate with the viral genome to act as a localization signal. Budding of the virion is formed through the assembly of several thousand copies of Gag lipid molecules in the host cell plasma membrane [9]. This process is finally completed when the spherical mature virion pinches off the inner side of the plasma membrane and carries its viral cargo, together with some extracellular fluid to its intracellular destination [10]. Alteration of HIV-1 protease activity using inhibitors has led to the deactivation of viral particles and reduced infectivity [11]. Without HIV-1 protease, the polyproteins would not be able to cleave into functional products and ultimately would not produce viruses from a host cell nor cause any infection. Therefore, due to the fundamental importance of HIV-1 protease for the realization of HIV, it has become the main target in the development of anti-HIV drug therapy [7].

HIV-1 protease inhibitors (Pls) approved by the United States Food and Drug Administration (US FDA) [12] include saquinavir, amprenavir, nelfinavir, indinavir, ritonavir, lopinavir, tipranavir, fosamprenavir, and darunavir [13, 14]. These inhibitors are substrate-based hydroxyethylamines that target the enzyme complex sites [15]. They are made up of an amino acid sequence of Serine-Lucine-Asparagine-Phenylalanine-Psi (CH(OH)-CH_2_N-Proline-Isoleucine-Methyl-LValinate, which binds the acetyl group (C_2_H_4_O) of HIV-1 protease [16]. Antiviral protease inhibitors mimic endogenous peptides by binding to the active site of HIV aspartyl protease, thereby preventing the cleavage of viral polyprotein precursors into mature functional proteins that replicate viral load [16, 17]. Combination of Pls with other inhibitors (e.g., the highly active antiretroviral therapy, HAART) with different mechanisms of action has led to a significant decline in HIV-1-associated morbidity and mortality [7, 18]. Long-term administration of HAART containing protease inhibitor has resulted in unpredicted adverse effects including hyperbilirubinaemia, insulin resistance, diabetes, and hyperlipidaemia [19]. Combinations of Pls without HAART could also have several detrimental side effects. Tipranavir co-administered with 200 mg of ritonavir led to (i) intracerebral hemorrhagic stroke, a devastating event that stems from the rupture of blood vessels in the brain, with the subsequent accumulation of blood in the parenchyma [20], and (ii) serious clinical hepatitis and hepatic infection [20–22]. Other side effects of Pls drugs are associated with diarrhea, nausea, rash, fever, fatigue, vomiting, abdominal pain, headache, bronchitis, depression, asthenia, insomnia, cough, diabetes mellitus, increased lipid levels, and changes in body fat levels [23]. The negative health effects of certain PIs and combinations thereof have led to an increased interest in alternative sources of antiprotease inhibitors.

*Carpobrotus edulis* (L.) belongs to the family of Aizoaceae, subfamily of Mesembryanthemaceae. The herb is indigenous to South Africa, and it is mostly found in the Eastern Cape Province, Northern Cape, KwaZulu-Natal, Free State, and Western Cape. It bears some common native names, including igukuma (Isixhosa), ikhambi-lamabulwo-umgongozi (IsiZulu), suurvy-rankvy-vyerank (Afrikaans), and freeway ice-plant (English) [24]. It is also commonly referred to as “Triple E,” i.e., “excellent edible evergreen” herb. *Carpobrotus edulis* (L.) is a perennial dense mat-forming herb with succulent leaves. The herb is resistant to drought and wind conditions and can be planted on flat, sandy ground and on loose sand dunes, gardens, lime-rich, brackish soils as well as in containers, rockeries, and embankments. The fruits can be eaten fresh, though they have a strong astringent, salty, and sour taste [25]. Local communities in the Eastern Cape Province traditionally use the herb's leaves as a remedy known to treat several infections including tuberculosis (TB), dysentery, diabetes, sore throat, and mouth infections. The roots and leaves are boiled in water or alcohol for 30–45 min, and approximately 5–125 ml was administered to adults and children depending on age and the severity of the illness. Leave extract powder is also prepared and used to treat burns, bruises, scrapes, cuts, ringworm, eczema, dermatitis, rash, and cracked lips. A mixture of leave, olive oil, and honey is used for the treatment of laryngitis and TB [26]. Previous studies have demonstrated the potential inhibitory effect of *C. edulis* extracts against several opportunistic infections common to HIV/AIDS [27–29]. The main phytochemical compounds found in hexane, acetone, and ethanol extracts, as determined with GC/MS, include monoterpenes, sesquiterpenes, diterpenes, phytol, and fatty acids with their methyl esters. These compounds were found to have high antioxidant, antifungal, and antimicrobial activities [27–29].

The present study evaluated the inhibitory effect of a water extract of *C. edulis* leaves against HIV-1 protease activity by utilizing a HiLyte Fluor™488/QXL™520 fluorescence resonance energy transfer (FRET) peptide as substrate. Cytotoxicity of the extract towards HeLa Chang cells was evaluated and the phytochemical profile determined with FT-IR and LC-MS.

## 2. Materials and Methods

### 2.1. Chemicals

Acetonitrile, formic acid, DMSO, and EDTA were purchased from Merck (Darmstadt, Germany); 3-(4,5-dimethylthiazol-2-yl)-2,5-diphenyl tetrazolium bromide (MTT) was purchased from Thermo Fisher Scientific (Cat no. M6494, AEC-Amersham SOC Ltd., South Africa); and curcumin (Cat no. C1386-5G) and potassium bromide (FT-IR grade; Cat no. 221864) were purchased from Sigma-Aldrich (Merck, Darmstadt, Germany). Water was successively purified by reverse osmosis followed by Milli-Q water purification (Millipore, Massachusetts, USA).

### 2.2. Collection of Plant Material

*Carpobrotus edulis* (L.) leaves were collected during late winter in August 2012 in the Nkonkobe Municipality district of the Eastern Cape Province of South Africa. The identity was confirmed by Prof. Grieson of the Department of Botany, University of Fort Hare (UFH), Alice, South Africa. A voucher specimen of the plant was preserved as “Omo 2012/1-Omo 2012/20” at the UFH herbarium. Leave samples were washed with distilled water, air-dried at room temperature for 14 days, and ground into a powder using a Romer Series II Grinding mill (Romer Labs, Newark, Delaware, USA).

### 2.3. Preparation of Plant Extract

Dried powdered leaves (100 g) were extracted with 1 L boiled distilled water on a mechanical shaker (Stuart Scientific Orbital Sharker 20.2, SOSI, Essex, UK) for 12 h. The extract was filtered through Whatman No 4 filter paper (Merck, Darmstadt, Germany) and the filtrate was refiltered with Whatman No 1 filter paper. The final filtrate was freeze-dried, yielding 6.2 g dried extract, and stored desiccated at 4°C prior to use. Stock solutions of 16, 1.6, 0.16, and 0.016 mg/ml were prepared in distilled water and used in experiments evaluating the inhibition of HIV-1 protease activity. A stock solution of 2 mg/ml was prepared in distilled water, and two-fold serial dilutions were prepared in Dulbecco's minimum essential medium (DMEM; Cat no. 11960069, Thermo Fisher Scientific, AEC-Amersham SOC Ltd., South Africa) to obtain working solutions of 1, 0.5, 0.25, 0.125, 0.062, 0.031, and 0.015 mg/ml, which were used to evaluate the cytotoxic properties of the plant extract.

### 2.4. HIV-1 Protease Activity Assay

HIV-1 protease activity was determined according to the method of AnaSpec, Fremont, California, USA, utilizing the SensoLyte® 520 HIV-1 protease fluorimetric assay kit (Cat no. AS-71147, AnaSpec, Fremont, California, USA) and black, flat-bottom 96-well microplates with nonbinding surfaces (Cat no. CLS3600, Merck, Darmstadt, Germany). Assay working solutions were freshly prepared by following the instructions with regards to the enzyme assay buffer, active recombinant HIV-1 protease stock solution (0.2 mg/ml; Cat no. 72028-5, AnaSpec, Fremont, California, USA), HIV-1 fluorescence resonance energy transfer (FRET) substrate, and the reference aspartic acid protease (pepstatin A) inhibitor.

#### 2.4.1. Optimization of HIV-1 Protease Activity

In order to determine the optimal enzyme activity response, the following HIV-1 protease combinations were evaluated: (i) 800-fold diluted HIV-1 protease stock in 1x enzyme buffer (40 *μ*l) and nuclease-free water (10 *μ*l), (ii) 80-fold diluted HIV-1 protease stock in 1x enzyme buffer (40 *μ*l) and nuclease-free water (10 *μ*l), and (iii) to evaluate the effect of the inhibitor, water was replaced with pepstatin A solution (10 *μ*l) in each reaction combination.

The enzymatic reactions were initiated by adding HIV-1 protease FRET substrate solution (50 *μ*l) and plates incubated at 25°C for 15 min. Reactions were performed in triplicate. The fluorescence intensity of the HIV-1 protease activity was measured at an excitation (Ex) wavelength of 490 nm and emission (Em) wavelength of 520 nm in minute intervals for 60 min using a fluorescence kinetic synergy MX analytical diagnostic reader (Gen spectrophotometer, Bio Tek, Vermont, USA). Data were transferred to a Microsoft Excel spreadsheet for further analysis. The average data generated were plotted against time.

#### 2.4.2. Determination of the Inhibitory Effect of the Plant Extract on HIV-1 Protease Activity

The following reaction combinations were prepared: (i) 80-fold diluted HIV-1 protease stock in 1x enzyme buffer (40 *μ*l) and plant extract (10 *μ*l) from each concentration (16, 1.6, 0.16, and 0.016 mg/ml), respectively, (ii) positive controls containing 80-fold diluted HIV-1 protease stock in 1x enzyme buffer (40 *μ*l) and nuclease-free water (10 *μ*l), and (iii) inhibitor controls containing 80-fold diluted HIV-1 protease stock in 1x enzyme buffer (40 *μ*l) and pepstatin A (10 *μ*l). The enzymatic reactions were initiated by adding HIV-1 protease FRET peptide substrate solution (50 *μ*l) and plates incubated at 25°C for 15 min. The reactions were performed in triplicate. Microplates were processed as described above. The percentage inhibition of each extract activity was calculated as

(1) $$\begin{array}{c} \%\text{ inhibition}=\frac{\text{HIV}‐1\text{ protease Abs}‐\text{extract Abs/positive control Abs}}{\text{HIV}‐1\text{ protease Abs}}\times 100\text{,} \end{array}$$

where Abs = absorbance. Graphs were generated by plotting inhibition percentage values against extract concentrations. The half maximal inhibitory concentration (IC_50_) was calculated from the percentage values.

### 2.5. MTT Cytotoxicity Assay

The MTT cytotoxicity assay utilizing HeLa Chang cells was used to calorimetrically assess the effect of the plant extract on viable cell metabolic activity [30]. MTT, a yellow dye, is reduced by cellular enzymes to the purple product, formazan. The reduction of the dye is proportional to the density of viable cells. Assays were performed in triplicate.

#### 2.5.1. Cell Line and Culture Medium

The HeLa Chang cell line used in this study was obtained from Prof. Van de Venter (Faculty of Medicine, Nelson Mandela Metropolitan University, South Africa). Cells were maintained in DMEM supplemented with 10% fetal bovine serum (Cat no. 16000044, Thermo Fisher Scientific, AEC-Amersham SOC Ltd., South Africa) and subcultured every two-three days [31]. At 80–90% confluence, cells were detached by adding Trypsin-EDTA solution [0.25% trypsin (w/v) in 1 mM EDTA, pH 7.4] and incubated for 3 min [32] at 37°C in a humidified incubator and 5% CO_2_ (Standard Model Heracell™ VIOS 250i, Cat no. 51030992, Thermo Fisher Scientific, AEC-Amersham SOC Ltd., South Africa).

#### 2.5.2. MTT Cytotoxicity Assay

The cytotoxicity assays were conducted in 96-well microplates (Cat no. 6905A11, Thermo Scientific™ Nunc™, Thermo Fisher Scientific, AEC-Amersham SOC Ltd., South Africa) by seeding at a density of 2000 cells/well with 100 *μ*l of suspension. Cells were treated with 100 *μ*l of 1, 0.5, 0.25, 0.125, 0.062, 0.031, and 0.015 mg/ml plant extract in DMEM working solutions, respectively. The following control combinations were included: (i) negative controls containing DMEM (100 *μ*l) and (ii) positive control containing 0.1 mg/ml curcumin (100 *μ*l). The microplates were incubated for 24, 48, and 72 h as described above. Following incubation, the medium was removed by aspiration and replaced with DMEM (100 *μ*l) and MTT (5 mg/ml in phosphate buffered saline, pH 7.4; 20 *μ*l) and further incubated for 4 h. Finally, the medium was aspirated and replaced with fresh DMSO (100 *μ*l) to solubilize the formazan crystals formed in the cells. The absorbance was measured at 570 nm with a fluorescence kinetic synergy MX analytical diagnostic reader (Gen spectrophotometer, Bio Tek, Vermont, USA).

### 2.6. Statistical Analysis

The NCSS 2019 software was used for statistical analysis [33]. Data were subjected to natural log (ln) transformation of all variables and analysed within a generalised linear model ANOVA. Multiple comparisons were analysed using the Tukey–Kramer multiple comparison procedure. This method provides joint simultaneous confidence intervals for all pairwise differences between the means and also provides the multiple comparison *p* value. Generally, *p* < 0.05 was used as statistical significance. In addition, the size of the F-ratios was used to measure relative sizes of differences.

### 2.7. Determination of the Phytochemical Profile with FT-IR Spectroscopy

Fourier-transform infrared (FT-IR) spectrophotometry is one of the most important analytical techniques used for identifying organic and inorganic chemical compounds and functional groups in plant samples. It can provide a snapshot of any metabolic composition of a tissue at a given time by measuring the infrared radiation of bonds within chemical functional groups and generating a spectrum that can be regarded as a biochemical fingerprint of the sample [34].

The *C. edulis* powdered water extract was analysed for characteristic functional groups using FT-IR spectrophotometry (Perkin Elmer Spectrum Series Model-2000, Japan). In brief, powdered extract (2 mg) and potassium bromide (2.5 mg) were homogenized using a mortar and pestle. A portion of the homogenized mixture was placed onto the FT-IR loading disc, pressed with a mini hand press to form a thin film of potassium bromide, and loaded into the FT-IR spectrophotometer with the scan range set at 4000 to 500 cm^−1^ and resolution at 4 cm^−1^. Identification of functional groups was analysed using the reference standard absorption peak which corresponds to bonds in the infrared region [34].

### 2.8. Identification of the Extracted Compounds with LC-MS

Liquid chromatography coupled to a mass spectrometer detector (LC-MS) was performed according to the methods of Akinrinde et al. [31], using a high performance Agilent 1260 Infinity Liquid Chromatography (LC) system, equipped with an Ab Sciex 5600 Triple TOF hybrid mass spectrometer (Applied Biosystems (AB) SCIEX, Framingham, MA, USA). The 5600 AB SCIEX Triple TOF has high selectivity and sensitivity accuracy (<5 ppm) that quantify and identify large panels of analytes across different compound classes in a single scan. This has made it valuable for small molecule analysis throughout the world [31]. Plant extract (2 mg/ml) in distilled water was directly analysed by LC-MS. The compounds were separated on a reversed-phase Proshell 120 EC-C18 column (4.6 × 50 mm; particle size 7 *μ*m) (Agilent InfinityLab LC, Stevens Creek Blvd, Santa Clara, USA). The mobile phase consisted of two solvents: solvent A—water with 0.1% formic acid and solvent B—acetonitrile with 0.1% formic acid. A linear gradient was followed from 75% solvent A to 25% solvent A over 15 min, ended by 2 min of column reequilibration at 75% solvent A. The injection volume was 5 *μ*l and the flow rate 1 ml/min.

TOF-MS parameters were as follows: ion spray voltage floating (ISVF) : 4500 V; ion source gas 1 (GS 1): 45 psi; ion source gas 2 (GS 2): 45 psi; and temperature (TEM): 450°C. Product ion parameters were as follows: the declustering potential (DP) was 60 V, while collision energy (CE) was set at 35°V. The spectra were recorded during ESI in negative mode at a mass range of m/z 50 and 1000. The acquisition was performed using Analyst Software and the data treatment, using XCMS online software (https://xcmsonline.scripps.edu) [35]. Hence, XCMS was used for preprocessing of all scanned data (3361 potential peaks) to extract potential molecular features, whose isotopic distribution was defined with peak spacing tolerance of m/z 0.01 and a mass accuracy of 10 ppm.

Ion formation during electrospray in negative mode using two ions (H^−^ and +C1^−^) was included to identify peaks with prospective metabolites. The resulting molecular ions were identified by examining the MS and MS/MS information in the Food Database (http://foodb.ca/) Version 1.0 (Canadian Institutes of Health Research (CIHR), Canada), with an error limit of 5 ppm. In addition, ChemSpider database software (Research Center, Raleigh, North Carolina, USA) was used to identify the molecular structure of each compound. Finally, the chemical structures of the compounds were drawn using ChemDraw 12.0 software, PerkinElmer (Perkin Elmer Ltd, Beaconsfield in Buckinghamshire, England).

## 3. Results and Discussion

### 3.1. Optimization of HIV-1 Protease Activity

Inhibition of HIV-1 protease activity is one of the primary aims in the development of antiviral agents for the treatment of HIV-infected patients [36]. The SensoLyte® 520 HIV-1 protease assay kit (Cat no. AS-71147, AnaSpec, Fremont, California, USA) provides a convenient assay for high throughput screening of HIV-1 protease inhibitors and continuous quantification of HIV-1 protease activity using a FRET peptide as substrate. The general sequence of the FRET peptide is derived from the native p17/p24 cleavage site on protease gag for HIV-1 protease. Incubation of recombinant HIV-1 protease with the fluorogenic substrate results in specific cleavage at the Tyr-Pro bond and a time-dependent increase in fluorescence intensity that is linearly related to the extent of substrate hydrolysis, which can be monitored at excitation and emission wavelengths of 490 nm and 520 nm, respectively. The fluorescence quantum yields of the HIV-1 protease substrate in the assay can increase up to 40.0- and 34.4-fold per mole of substrate cleaved.

**Figure 1:**
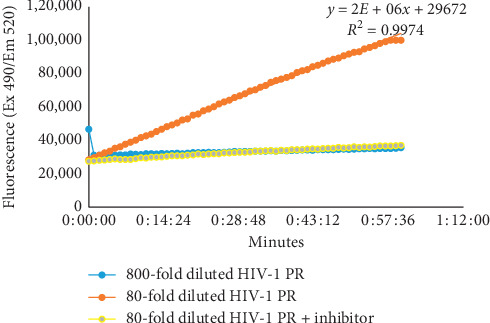
A kinetic linear progress graph presenting fluorescence intensity as a function of the rate of hydrolysis of the FRET peptide substrate over time in (i) 800-fold diluted HIV-1 protease stock (blue), (ii) 80-fold diluted HIV-1 protease stock (*R*^2^ = 0.9974) (orange), and (iii) 80-fold diluted HIV-1 stock and pepstatin A inhibitor (yellow).

To determine the optimal HIV-1 protease activity response concentration, the rate of substrate hydrolysis of 80- and 800-fold diluted HIV-1 protease stock (0.2 mg/ml) was evaluated. The 80-fold diluted stock yielded a time-dependant linear increase in fluorescence intensity, with a signal sufficient to measure inhibition, while the 800-fold dilution exhibited no fluorescence signal (Figure 1). The pepstatin A inhibitor effectively inhibited the 80-fold diluted HIV-1 protease.

### 3.2. Determination of the Inhibitory Effect of the Plant Extract on HIV-1 Protease Activity

**Figure 2:**
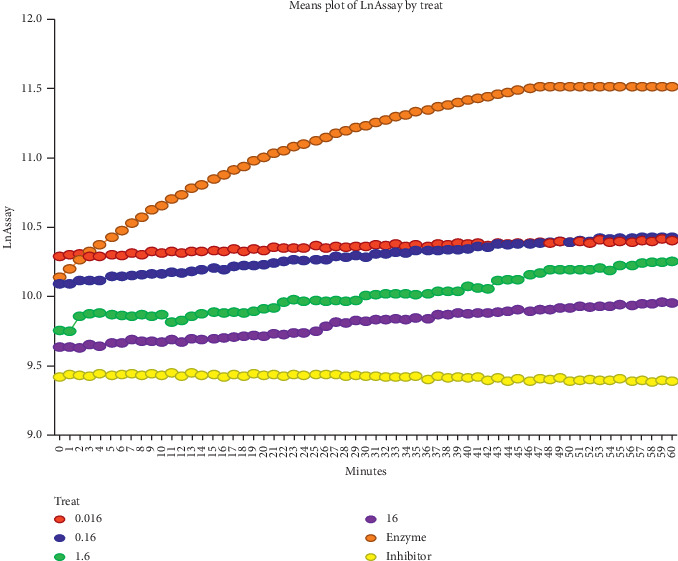
Treatment with all *C. edulis* extract concentrations inhibited HIV-1 protease activity at each time point during the linear curve (*p* < 0.0001). The observations show that most effective inhibition was 16 mg/ml (75%, IC_50_ 1.5 mg/ml) (*p* < 0.01).

**Figure 3:**
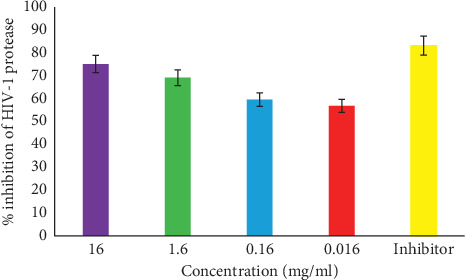
Percentage inhibition of HIV-1 protease activity by various concentrations. The inhibitor (pepstatin A) showed 83% inhibitory effect on the enzyme. Each extract concentration significantly (*p* < 0.05) inhibited HIV-1 protease activity >50%.

Medically, while the elusive cure to HIV/AID is still in progress, natural products from plants and their synthetic derivatives remain the alternative medicine for managing the disease. Some of these natural compounds that have demonstrated antiviral activities are found to inhibit vital enzymes and proteins critical to the life cycle of HIV, including the reverse transcriptase process, virus entry, integrase, and protease [37]. The extract of *C. edulis* used in the current study showed a remarkable anti-HIV enzyme activity against HIV-1 protease. The activity varied with respect to concentrations (16–0.016 mg/ml) against the 80-fold diluted HIV-1 protease, which exhibited a high substrate hydrolysis rate, following a linear trend over 60 minutes (Figure 2). The inhibitor control completely *p* < 0.0001) inhibited HIV-1 protease activity with 83.06% (IC_50_ 1.6 mg/ml). Treatment with all *C. edulis* extracts inhibited HIV-1 protease activity highly significant (*p* < 0.0001) at each time point during the linear curve (*R*^2^ = 0.9974) in a typical dose response manner. The most effective inhibition was observed with 16 mg/ml (75%, IC_50_ 1.5 mg/ml) extract, which was significantly (*p* < 0.01) more effective than the other extract concentrations (Figures 2 and 3). The 1.6 mg/ml treatment was significantly more effective (69%, IC_50_ 1.4 mg/ml) than the 0.16 mg·ml (59.5%, 1.2 mg/ml) treatment. There was no significant (*p* > 0.05) difference between the 0.016 and 0.16 mg/ml treatments. The plant extract treatments were slightly less effective (*p* > 0.05) than the inhibitor control.

Effective inhibition of HIV-1 protease activity by water extracts of several medicinal plant species has been reported worldwide. In South Africa, water extracts of *Cassia garrettiana* (94.6% inhibition; IC_50_ 2.0), *Hypoxis sobolifera*, and *Bulbine alooides* at 0.2 mg/ml (>50% inhibition; IC_50_ 1.19 mg/ml) inhibited HIV-1 protease [38]. Water extracts of 18 medicinal plants from China, Japan, and Indonesia inhibited HIV-1 protease activity >70% at concentrations between 25 and 250 *μ*g/ml [39]. Among the extracts examined, *Belamcanda chinensis*, *Magnolia fargesii*, *Paeonia suffruticosa*, *Phellodendron amurense*, and *Terminalia chebula* were the most effective at 25 *μ*g/ml. The Ayurvedic Indian medicinal plants *Areca catechu*, *Eugenia jambolana*, *Saraca indica, Adhatoda vasica*, and *Terminalia arjuna* exhibited >70% inhibition at 0.2 mg/ml (IC_50_ 1.22–1.4 mg/ml) [40, 41]. Water extracts of herbs indigenous to China, including *Geum japonicum*, *Punica granatum*, *Rhus javanica*, and *Woodwardia orientilis* exhibited >70% inhibition at 250 *μ*g/ml [42, 43]. Inhibition of HIV-1 protease activity by *Fuscoporia obliqua* harvested in Hokkaido, Japan, reduced enzyme activity by 50% at 2.5 *μ*g/ml [44]. In Hong Kong, water extracts of *Prunella vulgaris* and *Scutellaria baicalensis* exhibited 90% inhibition at 200 mg/ml [45], while treatment with *Paeonia suffruticosa* and *Mentha haplocalyx* at 20 mg/ml resulted in 40% inhibition. Water extracts of the Thai medicinal plant species *Dioscorea birmanica* (60 *μ*g/ml; 90% inhibition; IC_50_ 4.5 *μ*g/ml), *Smilax corbularia* (70 *μ*g/ml; >100%; IC_50_ 5.4 *μ*g/ml), and *Smilax glabra* (80%; >100%; IC_50_ 8.5 *μ*g/ml) exhibited effective inhibition [46]. *Rhus parviflora* Roxb. is a popular medicinal herb that is widely distributed in Nepal, Northern India, Bhutan, and Sri Lanka which is used for the treatment of neurological disorders, including anxiety, insomnia, epilepsy, and rheumatoid arthritis [47]. A water extract of *R. parviflora* inhibited HIV-1 protease activity in a dose-dependent manner (10, 20 and 50 *μ*g/ml) with maximum inhibition of >65% at 50 *μ*g/ml (IC_50_ 15 *μ*g/ml) [47]. *Acacia nilotica* and *Maytenus senegalensis*, medicinal plant species of Sudan, significantly (*p* < 0.05) inhibited HIV-1 protease both at 100 mg/ml with IC_50_ values of 48 mg/ml and 88 mg/ml, respectively. It should be noted that traditional healers do not prescribe organic solvent extracts of hexane or acetone to patients, rather they are conversant with natural solvents such as water or alcohol decoctions [28]. The toxicity of any medicinal plant should, however, be verified before administering to patients.

### 3.3. Cytotoxicity of *C. edulis* Water Extract

**Figure 4:**
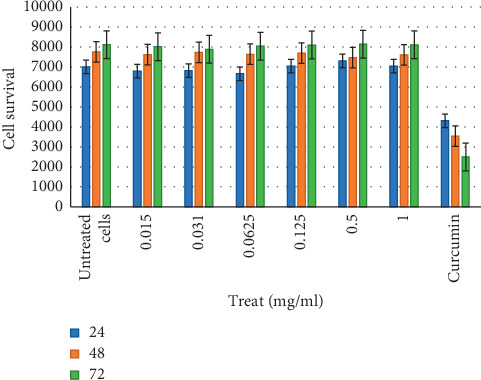
The cytotoxic effect of *C. edulis* water extract and curcumin (positive control) on HeLa Chang cells. Assays were performed in triplicate and results are expressed as the mean ± standard deviation of three independent experiments. Viable cells treated with different concentrations of the extract did not affect cell survival at 24, 48, and 72 h. Effect of curcumin monitored at same various hours was found toxic to the cells.

Scientific findings have shown that the majority of plant species used for medicinal purposes could be potentially toxic, mutagenic, and carcinogenic [48]. Researchers support the application of human cell lines for *in vitro* cytotoxicity assays to evaluate the toxicity of medicinal plants towards humans [49]. *Carpobrotus edulis* water extracts should be nontoxic or of low toxicity to human cells in order to be regarded as suitable for the development of preparations for the treatment of humans [49]. The negative controls containing untreated HeLa Chang cells exhibited high formazan formation rates during 72 h incubation in contrast with the positive controls, containing curcumin, which exhibited cytotoxicity towards the cells (Figure 4). There was no significant (*p* > 0.05) difference between the responses of the negative controls and 0.015, 0.031, 0.065, 0.125, 0.25, 0.5, and 1 mg/ml extract concentrations for all three time points, confirming no toxicity of *C. edulis* extracts towards HeLa Chang cells (Figure 4). Water extracts of medicinal plants with nonsignificant cytotoxicity include *Sclerocarya birrea,* a valued medicinal plant in South Africa, which possesses a variety of pharmacological attributes, including anti-inflammatory, antimicrobial, and antidiarrhea properties [50]. Due to its medicinal importance, water and acetone extracts were screened against various cancer cell lines, including HeLa Chang cells. Only the water extract was found nonsignificantly toxic to the cells [50]. A study by Sireeratawong et al. [51], revealed that water extract of *Hibiscus sabdariffa* showed weak toxicity in rats *in vivo* and was confirmed less harmful against HeLa Chang cell lines *in vitro*. Cytotoxicity evaluation of *Brachylaena elliptica* water extract also displayed low level of toxicity on HepG liver cells in all the doses tested [52].

### 3.4. Phytochemical Profile

**Figure 5:**
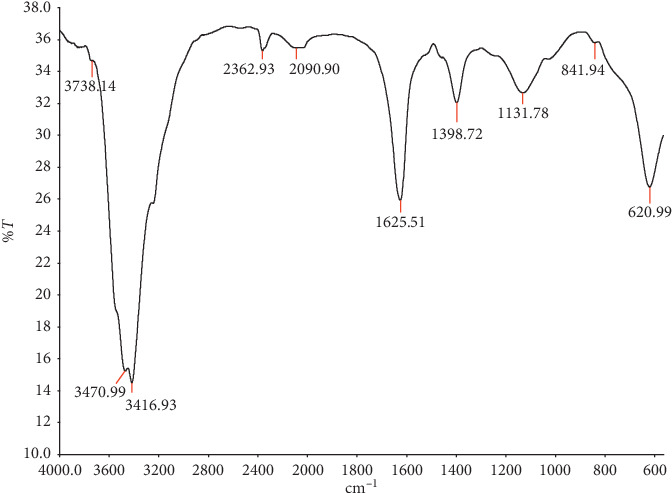
FT-IR spectrum of *C. edulis* water extract.

Fourier-transform infrared (FT-IR) spectrophotometer is regarded as the most accurate analytical technique for identifying chemical bonds and functional groups in plant compounds [53]. Chemical bonds together with their functional groups are determined from the highest to the lowest absorption band cm^−1^ peaks (Figure 5). The peak characteristic band at 3738 cm^−1^ shows the presence of H-O stretching, indicating alcohols and phenols; at 2362 cm^−1^, none was detected; the characteristic band at 2090 cm^−1^ shows the -C≡C- bending, indicating alkynes; the characteristic band at 1625 cm^−1^ shows N-H stretching amines; the characteristic band at 1398 cm^−1^ shows C-O-H bending, indicating presence of alcohols; the characteristic band at 1131 cm^−1^ shows C-O stretching carboxylic acids and esters; the characteristic band at 814 cm^−1^ shows out of plane, meaning none was detected; and the characteristic band at 620 cm^−1^ shows C-H group, indicating the presence of alkanes (Table 1).

Phenols and alcohols, such as quercetin and flavonoids, are reported to have anti-inflammatory, antioxidant, and free radical scavenger properties [54]. In the past, phenol was the first antiseptic used in surgery. Anticancer activity of green tea cannabinoids is currently being used in cancer patients to palliate wasting, emesis, and pain that often accompany cancer [55]. The fruit aroma of pineapples, pears, and strawberries are caused by esters [56], which are applied as anesthetic [57]. Benzocaine, a carboxylic acid, is used to reduce dental pain [58]. Compounds containing benzene rings are used by the pharmaceutical industries to produce drugs, such as ibuprofen, aspirin, acetaminophen (tylenol), amphetamine, and sulphanilamide (NSAIDS). These drugs are used for toothache, back pain, menstrual cramps, muscle pain, pain from arthritis, fever, and the prevention of heart attacks or stroke (NSAIDS).

### 3.5. Identification of the Extracted Compounds by LC-ESI-TOF/MS

**Figure 6:**
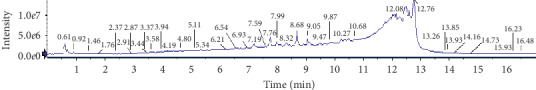
ESl-MS total ion chromatogram of the aqueous extract of *C. edulis* during negative ionization mode.

**Figure 7:**
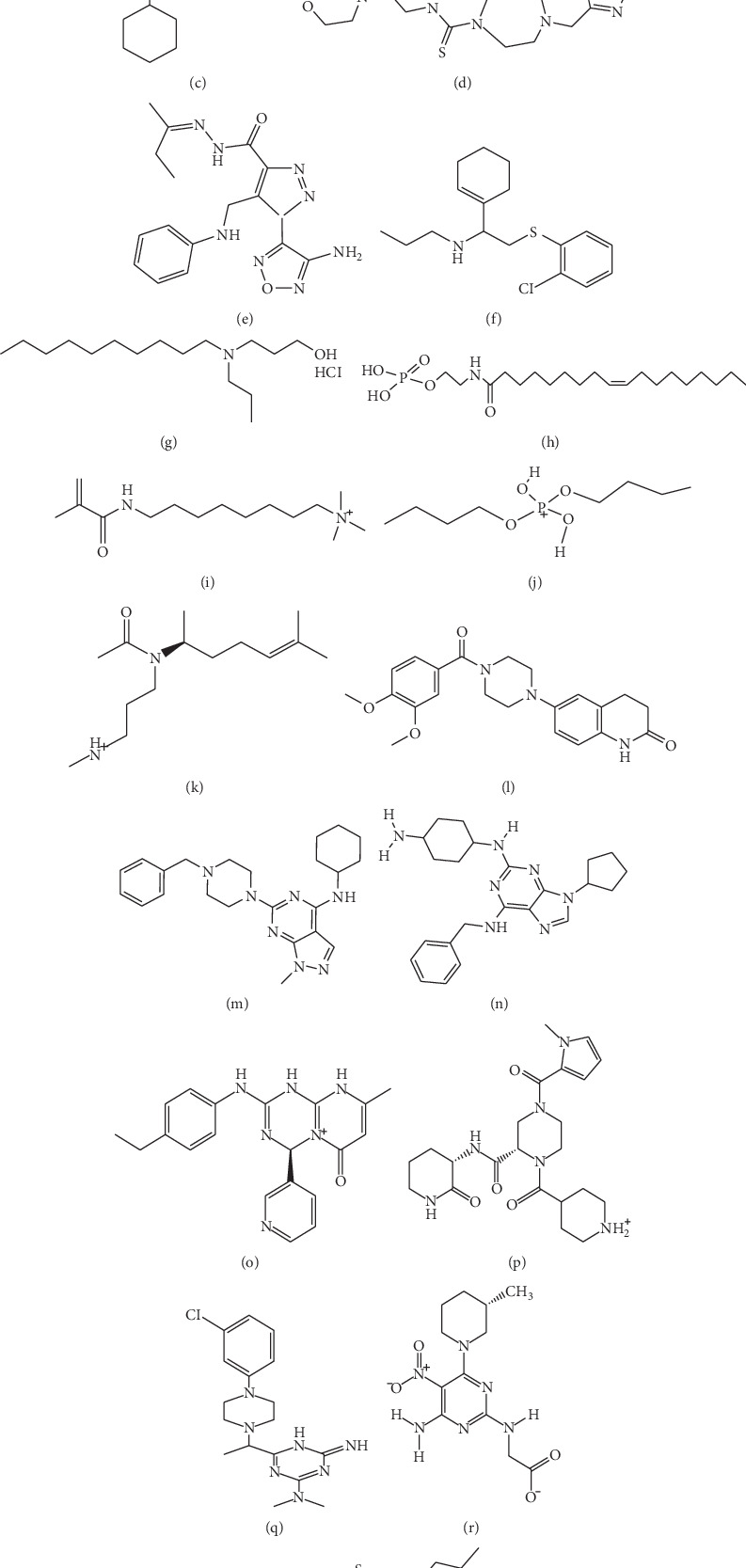
Chemical structures of main identified compounds. (a) Di-ethoxydimethylphosphoramide. (b) Dimethyl-2-(2-nitrophenoxy)ethyl-azanium. (c) N,N′-Dicyclohexyl-4-morpholinecarboxamidine. (d) N-(2-Morpholin-4-ylethyl)-4-(1,3-thiazol 2-ylmethyl)-1,4-diazepane-1-carbothioamide. (e) 1-(4-Amino-1,2,5-oxadiazol-3-yl)-N-(4-dimethylaminophenyl)methylideneamino-5-ethyltriazole-4-carboxamide. (f) N-2-(2-Chlorophenyl)sulfanyl-1-(1-cyclohexen-1-yl)ethyl-1-propanamine. (g) 2-Hydroxyethyl(tetradecan-2-yl) azanium chloride. (h) 4-(Z)-Hexadec-9-enoyl-amino-butyl dihydrogen phosphate. (i) 3-Acetyl-(2R)-6-methylhept-5-en-2-yl-amino-propyl-dimethylazanium. (j) Dibutoxy(dihydroxy)phosphanium. (k) 2-Octyl-1-cyclopropene-1-octanoate (sterculate). (l) Vesnarinone. (m) 2-N-(4-Aminocyclohexyl)-6-N-benzyl-9-cyclopentylpurine-2,6-diamine. (n) 2-trans-(4-Aminocyclohexyl) amino-6-(benzyl-amino)-9-cyclopentylpurine. (o) Amino-(4-benzamidoanilino) methylidene-(4,6 dimethylpyrimidin-2-yl) azanium. (p) (2S)-4-(1-Methylpyrrole-2-carbonyl)-N-(3S)-2-oxopiperidin-3-yl]-1-(piperidin-1-ium-4-carbonyl) piperazine-2-carboxamide. (q) N-(3-Chloro-4-methylphenyl)-6-(4-ethyl-1-piperazinyl) methyl-1,3,5-triazine-2,4-diamine. (r) Vitamin B. (s) 1R,9aR-1,2,3,4,5,6,7,8,9,9a-Decahydroquinolizin-5-ium-1-yl-methoxy-methyl-pentoxy-sulfanylidene-lambda-5-phosphane-chloride.

HIV-1 protease inhibitors (pls) are regulatory proteins found in numerous animal tissues and plants that reduce and inhibit proteases involved in human diseases like arthritis, pancreatitis, hepatitis, cancer, AIDS, thrombosis, emphysema, hypertension, and muscular dystrophy among others, thus pointing to possible applications in biomedicine, agriculture, and biotechnology [41]. The full scan acquisition of each compound peak was analysed using the peak view Analyst Software (ABSciex) [59]. The MS total ion chromatogram obtained from the analysis scanned in negative mode is shown in Figure 6. The negative mode was selected based on its sensitivity in detecting polyphenols, especially phenolic acids, flavonoids, and alkaloids. Chemical compounds present in the extract were identified using the XCMS software, followed by ChemSpider database software.  Table 2 presents the class of compounds identified as [M-H]^−^ molecular ions and m/z values. Structure assignment of the various molecular ions analysed is presented in Figure 7. Our discussion focuses on the identification of the most interesting compounds with anti-inflammatory activities.

In total, 8 phenolic acids were tentatively identified, including 2 flavonoids. Of these, 1 flavonoid and 1 phenolic acid were identified as the [M-P]^−^. These were Di-ethoxydimethylphosphoramide (C_6_H_18_N_3_O_3_P) at m/z 211.108 and 4-(Z)-hexadec-9-enoyl-amino-butyl dihydrogen phosphate (C_20_H_40_NO_5_P) at m/z 211.108. In addition, five ions were identified as [M-H]^−^, including dimethyl-2-(2-nitrophenoxy)ethyl-azanium (m/z211.107); N,N′-dicyclohexyl-4-morpholinecarboxamidine (m/z 293.246); N-(2-morpholin-4-ylethyl)-4-(1,3-thiazol 2-ylmethyl)-1,4-diazepane-1-carbothioamide (m/z 369.165); 1-(4-amino-1,2,5-oxadiazol-3-yl)-N-(4-dimethylaminophenyl)methylideneamino-5ethyltriazole-4 carboxamide (m/z 309.131); and 2-hydroxyethyl(tetradecan-2-yl) azanium-chloride (m/z 293.253). Only N-2-(2-chlorophenyl) sulfanyl-1-(1-cyclohexen-1-yl) ethyl-1-propanamine (M-C_17_H_24_NOCI) was identified at retention time (RT) 6.5. Several polyphenolic derivatives, for example, resveratrol, tocopherols, phytosterols, and carotenoids, detected by the formation of [M-H]^−^ ions are known to possess antioxidants protecting the cells of the body from oxidative damage [31]. They provide a range of activities from inhibiting cancer cell proliferation to protecting the immune system against cardiovascular disease [31]. Evaluation of the anti-influenza activity of 1*H*-1,2,3-triazole-4-carboxamide derivatives at [M-H]^−^ was found to inhibit the replication of virus strains, including H5N1 (RG14), amantadine-resistant A/WSN/33 (H1N1), and oseltamivir-resistant A/WSN/1933 (H1N1, 274Y) [60]. Antiproliferative activities of alpha-viniferin detected at m/z 677.1817 and hyperoside at m/z 463.0882 M-H^−^ were reported to reduce the growth of the human heptocellular carcinoma (HepG2) cells and murine insulinoma (INS-1) cells [31]. Isoacteoside detected by the formation of the [M + CI]^−^ at RT 8.35 was observed to have anticancer property [31].

Generally, phytochemicals are classified into six major categories based on their chemical structures and characteristics. These categories include carbohydrate, lipids, phenolics, terpenoids and alkaloids, and other nitrogen-containing compounds. Lipids serve five major functions in living organisms: they act as energy source, provide insulation and protection to organs, give structure to cell membranes, and generate heat when temperature changes [42]. Most people have enough lipids that act as a food source for 24 to 30 days [42]. About 4 important lipids were detected at the base peak of [M-H]^−^ at different retention times, including 3-acetyl-(2R)-6-methylhept-5-en-2-yl-amino-propyl-dimethylazanium (m/z 255.243), dibutoxy(dihydroxy)phosphanium (m/z 405.262); 2-octyl-1-cyclopropene-1-octanoate (sterculate) (m/z 293.248), and vesnarinone (m/z 405.54). Sterculates are vital natural compounds found in many plant species belonging to *Sterculiaceae*, *Malvaceae*, *Tiliaceae*, and *Bombacaceae* families [61]. They are majorly used as parasitic drugs to control insect and fungi infestation in plants [61]. Congestion of heart failure is a common problem associated with prognosis. Patients with an advanced stage of the disease are treated with vesnarinone [62]. The compound is a quinolinone derivative, a small molecule with phosphodiesterase 3 (PDE 3) inhibitor [63]. *In vit*ro studies have shown that vesnarinone suppresses the production of TNF-*α* and IL-6 in various human cell lines, including peripheral lymphocytes, monocytes, and T-cell lines [64]. It was also found to suppress cell proliferation and induces apoptosis on the expression of p21 in p53-mediated cell cycle [64]. Currently, a clinical trial of vesnarinone is being tested on patients with Kaposi sarcoma associated with HIV at the National Institute of Health (NIH).

Other compounds, belonging to alkaloids and nitrogen-containing compounds, were tentatively identified which reported antiproliferative activities. Two purine derivatives of 2-N-(4-aminocyclohexyl)-6-N-benzyl-9-cyclopentylpurine-2,6-diamine ([M-H]^−^ at m/z 405.263) and 2-trans-(4-aminocyclohexyl) amino-6-(benzyl-amino)-9-cyclopentylpurine ([M-H]^−^ at m/z 405.263) were identified at retention times of 12.5 min and 7.0 min, respectively. The number of pyrimidine derivatives identified at base peaks of [M-H]^−^ was two and one chlorine-ion [M + Cl]^−^, including amino-(4-benzamidoanilino) methylidene-(4,6 dimethylpyrimidin-2-yl) azanium (m/z 361.177); (2S)-4-(1-methylpyrrole-2-carbonyl)-N-(3S)-2-oxopiperidin-3-yl]-1-(piperidin-1-ium-4-carbonyl) piperazine-2-carboxamide (m/z 445.255); and N-(3-chloro-4-methylphenyl)-6-(4-ethyl-1-piperazinyl) methyl-1,3,5-triazine-2,4-diamine (m/z 293.247-C_17_H_24_N_7_CI). A pronounced vitamin B-C_2_H_17_N_6_O_4_ was observed at m/z 309.130 (RT 8.0 min) and a quinolinone derivative of 1R,9aR-1,2,3,4,5,6,7,8,9,9a-decahydroquinolizin-5-ium-1-yl-methoxy-methyl-pentoxy-sulfanylidene-lambda-5-phosphane-chloride [M-C_16_H_33_NO_2_PSCI] was observed at 10.0 min retention time. Purines and pyrimidine derivatives are essential building blocks of nucleic acids, DNA, and RNA. They are used to produce drugs that treat multiple cancers, respiratory inflammations, and HIV/AIDS [65]. The drugs are also recommended for preterm labor, premature birth, and for controlling labor prior to caesarean delivery [66]. B vitamins are especially important for women who are pregnant and breastfeeding. The vitamins boost energy levels, ease nausea, aid in fetal brain development, and reduce the risk of birth defects. A deficiency of vitamin B1 (thiamine) may cause severe neurological symptoms.

## 4. Conclusions

The current study showed the *in vitro* anti-HIV enzyme activity of *C. edulis* aqueous leave extract. Water extract of *C. edulis* highly significantly (*P* < 0.0001) inhibits HIV-1 protease activity with no cytotoxic effect on HeLa Chang cells. Profiling of compounds present in the extract by LC-TOF-MS tentatively revealed a total of 19 compounds, mainly on the basis of accurate mass measurements of the metabolites. Purines and pyrimidine derivatives are compounds with reported anti-HIV activities. However, a second phase of study involving more detailed biological activities of each compound inhibition on the enzyme must be performed. Our current work and study will lead to follow a more specific route to better understand the activity of extract fraction.

## Figures and Tables

**Table 1:** Identified FT-IR peak values, functional groups, and compounds of *C. edulis* water extract.

Identified peak values (cm^−1^)	Functional groups	Nature of compounds
3738–3416	O-H	Alcohols, phenols
2362	ND	ND
2090	-C≡C-	Alkynes
1625	N-H	Amines
1398	C-O-H	Alcohols
1131	C-O	Carboxylic acids, esters
841	ND	ND
620	C-H	Alkanes

ND: none detected.

**Table 2:** Putative identification of compounds in *C. edulis* showing the main parameters for their identification.

Class of compounds	Putative identification	Mass	m/z	Adduct	RT (min)	*δ* (ppm)	Formula
Flavonoids	Di-ethoxydimethylphosphoramide	211.202	211.108	[M-P]^−^	0.5	3.2	C_6_H_18_N_3_O_3_P
	Dimethyl-2-(2-nitrophenoxy)ethyl-azanium	211.241	211.107	[M-H]^−^	0.5	3.2	C_10_H_15_N_2_O_3_

Phenolic acids	N,N′-Dicyclohexyl-4-morpholinecarboxamidine	293.455	293.246	[M-H]^−^	14.0	1.7	C_17_H_31_N_3_O
	N-(2-Morpholin-4-ylethyl)-4-(1,3-thiazol 2-ylmethyl)-1,4-diazepane-1-carbothioamide	369.546	369.165	[M-H]^−^	4.5	3.2	C_16_H_10_N_9_O_2_
	1-(4-Amino-1,2,5-oxadiazol-3-yl)-N-(4-dimethylaminophenyl)methylideneamino-5-ethyltriazole-4-carboxamide	369.389	309.131	[M-H]^−^	1.5	5.0	C_16_H_19_N_9_O_2_
	N-2-(2-Chlorophenyl)sulfanyl-1-(1-cyclohexen-1-yl)ethyl-1-propanamine	309.896	361.176	[M + Cl]^−^	6.5	0.2	C_17_H_24_NOCl
	2-Hydroxyethyl(tetradecan-2-yl) azanium-chloride	293.92	293.253	[M-H]^−^	−0.5	−2.5	C_19_H_33_O_2_
	4-(Z)-Hexadec-9-enoyl-amino-butyl dihydrogen phosphate	405.516	361.177	[M-P]^−^	2.5	−2.1	C_20_H_40_NO_5_P

Lipid derivatives	3-Acetyl-(2R)-6-methylhept-5-en-2-yl-amino-propyl-dimethylazanium	255.426	255.243	[M-H]^−^	5.0	4.6	C_15_H_31_NO_2_
	Dibutoxy(dihydroxy)phosphanium	211.218	405.262	[M-P]^−^	0.5	2.2	C_8_H_20_O_4_P
	2-Octyl-1-cyclopropene-1-octanoate (Sterculate)	293.471	293.248	[M-H]^−^	4.0	−1.4	C_19_H_33_O_2_
	Vesnarinone	405.53	405.54	[M-H]^−^	1.5	5.0	C_22_H_35_N_3_O_4_

Purine derivatives	2-N-(4-Aminocyclohexyl)-6-N-benzyl-9-cyclopentylpurine-2,6-diamine	405.55	405.263	[M-H]^−^	12.5	−1.3	C_23_H_31_N_7_
	2-trans-(4-Aminocyclohexyl) amino-6-(benzyl-amino)-9-cyclopentylpurine	405.55	405.263	[M-H]^−^	7.0	−1.4	C_24_H_37_O_5_

Pyrimidine derivatives	Amino-(4-benzamidoanilino) methylidene-(4,6 dimethylpyrimidin-2-yl) azanium	361.429	361.177	[M-H]^−^	7.5	2.0	C_20_H_21_N_6_O
	(2S)-4-(1-Methylpyrrole-2-carbonyl)-N-(3S)-2-oxopiperidin-3-yl]-1-(piperidin-1-ium-4-carbonyl) piperazine-2-carboxamide	445.544	445.255	[M-H]^−^	2.0	−0.4	C_22_H_33_N_6_O_4_
	N-(3-Chloro-4-methylphenyl)-6-(4-ethyl-1-piperazinyl) methyl-1,3,5-triazine-2,4-diamine	361.878	293.247	[M + Cl]^−^	9.5	0.4	C_17_H_24_N_7_Cl

Vitamin B	2-4-Amino-6-(3S)-3-methylpiperidin-1-yl-5-nitropyrimidin-2-yl-amino-acetate	309.306	309.130	[M-H]^−^	8.0	2.3	C_2_H_17_N_6_O_4_

Quinolinone derivative	1R,9aR-1,2,3,4,5,6,7,8,9,9a-Decahydroquinolizin-5-ium-1-yl-methoxy-methyl-pentoxy-sulfanylidene-lambda-5-phosphane-chloride	405.539	405.262	[M + CI]^−^	10.0	0.9	C_16_H_33_NO_2_PSCl

## Data Availability

All data generated or analysed during this study are included within the article.
